# Characteristics of Patients Undergoing Bariatric Surgery in an Inner-City Minority Population: A Pre and Postoperative Comparison Between Patients With and Without Obstructive Sleep Apnea

**DOI:** 10.7759/cureus.66440

**Published:** 2024-08-08

**Authors:** Abhishrut Jog, Jorge Mosquera Zavaleta, Luis Rodriguez Piedra, Ajit Singh, David Fan, Vincent Grbach, Dmitry Lvovsky

**Affiliations:** 1 Pulmonary Medicine, BronxCare Health System, Bronx, USA; 2 General Surgery, BronxCare Health System, Bronx, USA; 3 Sleep Medicine, Mount Sinai Hospital, New York, USA

**Keywords:** obstructive sleep apnea (osa), urban population, gastric weight loss surgery, hispanic population, minority population, new york city, postoperative outcomes, weight loss, health disparity population, bariatric surgery

## Abstract

Background

Obese patients are at an increased risk of obstructive sleep apnea (OSA). Bariatric surgery or weight loss surgery is an important therapeutic measure in obese patients for the management of weight and comorbidities. Data are scarce in inner-city Hispanic and Black patients who undergo bariatric surgery, which eventually leads to health disparity in this minority population. Differences between patients with and without OSA have not been assessed in this population. This study aims to answer these questions.

Methodology

The study was conducted in a high-volume hospital in the Bronx, New York. Before bariatric surgery, patients underwent a preoperative evaluation that included a variety of blood tests, a sleep study, esophagogastroduodenoscopy, and echocardiography. They also underwent basic anthropometric measurements, such as weight, height, and body mass index (BMI), before surgery and 6 months and 12 months postoperatively. Additional calculations were made using these anthropometric measures, namely, total weight loss, excess weight loss, and delta BMI.

Results

Most patients were Hispanic (85.2%), with a mean age of 41.9 ± 10.8 years. We found that of the 108 patients included in the study, 69.4% (70/108) had OSA. Preoperative BMI in the study was 43.9 ± 13 kg/m^2^. Postoperatively, the mean decrease in BMI was 12.3 ± 14.5 kg/m^2^. Total weight loss and excess weight loss were 30.2 ± 14.3 and 52.6 ± 16.6, respectively.

Conclusions

In this study, no significant difference was noted in patients with or without OSA in either the laboratory or anthropometric parameters.

## Introduction

In a study of the National Health and Nutrition Examination Survey (NHANES) database, done between the years 1999-2000 and 2017-2018, Ogden et al. showed that over the last two decades, the prevalence of obesity has increased among men and women in the United States (27.5% to 43.0% in men and 33.4% to 41.9% in women). The study also showed that there was a greater increase in obesity in Mexican American men than in non-Hispanic White men (3.0 (95% confidence interval (CI) = 2.4-3.6) vs. 1.4 (95% CI = 0.9-1.9) percent points bi-annually, p < 0.001) [1]. Obesity remains the strongest risk factor for the development of obstructive sleep apnea (OSA), and both diseases increase cardiovascular risk [2]. Bariatric surgery is now recognized as an important option for permanent and sustainable weight loss [3].

There is a scarcity of data available on Hispanic patients undergoing bariatric surgery. Zhao et al., in their meta-analysis, found a total of 52 studies on the topic of postoperative weight loss comparing at least two races. Of these 52 studies, only 20 included Hispanic patients. Some of these studies had less than 10% Hispanic representation, going as low as 4.7%. In addition, many studies had less than a year of follow-up [4].

To address this data vacuum, the following study was undertaken. In New York City, the Burrough with the highest prevalence of obesity is the Bronx (30.5%) [5]. The Burrough with the highest number of Hispanic and Black people is also the Bronx. As a result, the hospital where the study was conducted has a large pool of inner-city Hispanic or Black patients. This allowed an analysis of outcomes and characteristics in this underserved and understudied population.

This article was previously posted to the Research Square preprint server on June 4, 2024.

## Materials and methods

Study objectives

The study aimed to determine the various characteristics of patients qualifying for bariatric surgery, including anthropometric measures and laboratory tests. It also aimed to find out if there existed any significant difference between bariatric candidates with and without OSA based on these findings. Finally, the study aimed to analyze the postoperative outcomes of bariatric surgery in these patients at six months and one year with regard to weight loss.

Data collection

This was a single-center, multi-surgeon, retrospective study. The study protocol was approved by the Institutional Review Board of BronxCare Hospital (approval number: 04132305). All patients over the age of 18 years who underwent bariatric surgery from January 2021 to February 2023 at BronxCare Hospital System, Icahn School of Medicine at Mount Sinai, New York, were considered for inclusion in the study. Data were collected retrospectively by electronic medical record review.

Preoperative data

Pertinent information collected included demographics, sleep study findings (apnea-hypopnea index, minimum oxygen saturation, duration of desaturation), type of bariatric procedure, comorbidities (hypertension (HTN), type 2 diabetes mellitus (T2DM), coronary artery disease (CAD) cerebrovascular accident (CVA), atrial fibrillation (Afib), major depressive disorder (MDD)), and preoperative anthropometric measurements (weight measured in kilograms, and body mass index (BMI)). Preoperatively, certain laboratory parameters were collected, including glycated hemoglobin (HbA1C), serum cortisol, serum thyroid-stimulating hormone (TSH), and serum vitamin D. Included patients underwent preoperative echocardiography and data on the ejection fraction (EF) and pulmonary artery systolic pressure (PASP) were collected. Included patients also underwent preoperative esophagogastroduodenoscopy (EGD). Data were collected on the EGD findings and *Helicobacter pylori* testing using the rapid urease test (CLO test: *Campylobacter*-like organism test).

Postoperative data

Postoperative anthropometric measurements of weight and BMI were noted at 6 and 12 months.

Calculations

The following six parameters were calculated based on the pre and postoperative anthropometric measurements: excess weight loss % at six months (EWL6), excess weight loss % at 12 months (EWL12) as per equation (1), total weight loss % at six months (TWL6), total weight loss % at 12 months (TWL12) as per equation (2), Change in BMI % at six months (delta BMI 6), and change in BMI % at 12 months (Delta BMI 12) as per equation (3).

Equation 1: Excess weight loss: EWL = preoperative weight - postoperative weight (at 6 or 12 months) × 100/preoperative excess weight.

Equation 2: Total weight loss: TWL = preoperative weight - postoperative weight (at 6 or 12 months) × 100/preoperative weight.

Equation 3: Change in BMI: delta BMI = preoperative BMI - postoperative BMI (at 6 or 12 months) × 100/preoperative BMI.

Statistical analysis

Data were analyzed using STATA (StataCorp., College Station, TX, USA). Mean values were calculated for all numerical data. The chi-square test was applied to categorical variables. The t-test was applied for continuous variables. A p-value of less than 0.05 was considered significant.

## Results

**Figure 1:**
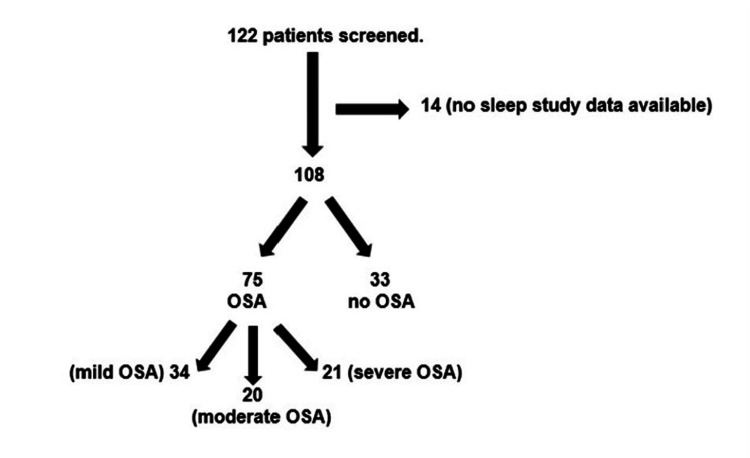
Distribution of patients. Out of 122 patients who underwent bariatric surgery, 14 patients had no sleep study. Finally, 108 patients were included in the final analysis, of whom 75 had OSA and 33 did not. Severe, moderate, and mild OSA was seen in 34, 20, and 21 patients, respectively. OSA = obstructive sleep apnea

A total of 122 bariatric surgeries were performed at the hospital in the study period. In total, 108 patients out of the screened patients had undergone a preoperative sleep study. Overall, 69.4% had OSA (75/108) and 30.6% (33/108) patients had no OSA. Further, 45.3% (34/75) had mild OSA, 26.7% (20/75) had moderate OSA, and 28% (21/75) had severe OSA. Figure 1 shows the distribution of patients.

Preoperative weight data was not available for one patient in the OSA group. Six-month postoperative weight data was not available for five patients in the OSA group. Moreover, 12-month postoperative weight data was not available for 21 patients in the OSA group and seven patients in the non-OSA group. Analysis of anthropometric measures was accordingly done on the available data. Overall, 88% of the participants were female (99/108). The mean age in the cohort was 41.9 ± 10.8 years. Further, 85.2% (92/108) of the total patients were Hispanic, and 14% (15/108) were Black. There were no active smokers (15.7% were former smokers and 84.3% were never smokers).

The mean preoperative weight was 111.2 ± 20 kg in those without OSA and 115.9 ± 22.6 kg in those with OSA. The mean preoperative BMI was 42.3 ± 6.6 kg/m^2^ in the group without OSA and 44.5 ± 15 kg/m^2^ in the group with OSA. In the group without OSA, the respective TWL6 and TWL12 were 26.7 ± 8.5 and 28.3 ± 9.2, and in the group with OSA, the respective TWL6 and TWL12 were 26.8 ± 13.6 and 31.1 ± 16.2 (p-value for TWL6 was 0.9 and for TWL12 was 0.4). In the group without OSA, the respective EWL6 and EWL12 were 49.4 ± 14.3 and 54.7 ± 18.4, and in the group with OSA, the respective values of EWL6 and EWL12 were 44.9 ± 13.9 and 51.7 ± 15.7 (p-value for EWL6 was 0.1 and for EWL12 was 0.5). In the group without OSA, the respective delta BMI 6 and delta BMI 12 were 10.11 ± 3.7 and 10.8 ± 4.4, and in the group with OSA, the respective values of Delta BMI 6 and Delta BMI 12 were 11.2 ± 15.3 and 13.1 ± 17.3 (p-value for Delta BMI 6 was 0.7 and for Delta BMI 12 was 0.5). Between the group without OSA and with OSA, no statistically significant difference was noted in the mean HbA1C (6.2 ± 1.1 vs. 6.1 ± 1.1, p = 0.9), TSH (1.85 ± 1 vs. 2.3 ± 2.3), serum cortisol (1.4 ± 2.3 vs. 1.1 ± 2.3, p = 0.7), vitamin D (18.1 ± 6.5 vs. 21.4 ± 9.2, p = 0.07), EF (65% ± 5.9 vs. 65% ± 6, p = 0.8), PASP (36.8 ± 9 vs. 40.2 ± 8.3, p = 0.1), and CLO test (45.5% vs. 43%, p = 0.8). Abnormal EGD findings were seen in 54.6% (59/108) of patients. In descending frequencies, the abnormal EGD findings were mucosal erythema in 29.6% (32/108), gastritis in 11.1% (12/108), hiatal hernia in 4.6% (5/108), gastric ulcer in 3.7% (4/108), gastric polyp in 2.7% (3/108), and esophagitis in 2.7% (3/108). Active MDD requiring medication was seen in 7.4% (8/108) of patients.

The results are presented below in Table 1. The types of surgeries performed are presented in Table 2.

**Table 1:** Study findings. The mean values with the standard errors are provided in the table. The chi-square test was applied to categorical variables. The t-test was applied for continuous variables. A p-value of less than 0.05 was considered significant. The test used to generate the p-value is shown in parentheses next to each p-value. c = chi-square test; t = t-test; BMI = body mass index; TWL = total weight loss; EWL = excess weight loss; HbA1C = glycated hemoglobin; TSH = thyroid-stimulating hormone; HTN = hypertension; T2DM = type 2 diabetes mellitus; CVA = cerebrovascular accident; CAD = coronary artery disease; Afib = atrial fibrillation; EF = ejection fraction; PASP = pulmonary artery systolic pressure; CLO = *Campylobacter*-like organism test; EGD = esophagogastroduodenoscopy

	No OSA (n = 33)	OSA (n = 75)	Total (n = 108)	P-value
Age (years)	39 ± 11.5	43.3 ± 10.3	41.9 ± 10.8	0.05 (t)
Male	1 (3%)	12 (16%)	13 (12%)	0.06 (c)
Female	32 (97%)	63 (84%)	95 (88%)	0.06 (c)
Hispanic	29 (88%)	63 (84%)	92 (85.2%)	-
Black	3 (9%)	12 (16%)	15 (14%)	-
White	1 (3%)	0	1 (0.8%)	-
Former smoker	6 (18%)	12 (16%)	18 (16.7%)	-
Never smoker	27 (82%)	63 (84%)	90 (83.3%)	-
Preoperative weight (kg)	111.2 ± 20	115.9 ± 22.6 (n = 74)	114.7 ± 21.6 (n = 107)	0.3 (t)
Preoperative BMI (kg/m^2^)	42.3 ± 6.6	44.5 ± 15 (n = 74)	43.9 ± 13 (n = 107)	0.4 (t)
TWL6	26.7 ± 8.5	26.8 ± 13.6 (n = 70)	26.8 ± 12.2 (n = 103)	0.9 (t)
TWL12	28.3 ± 9.2 (n = 26)	31.1 ± 16.2 (n = 54)	30.2 ± 14.3 (n= 80)	0.4 (t)
EWL6	49.4 ± 14.3	44.9 ± 13.9 (n = 70)	46.4 ± 14.1 (n = 103)	0.1 (t)
EWL12	54.7 ± 18.4 (n = 26)	51.7 ± 15.7 (n = 54)	52.6 ± 16.6 (n= 80)	0.5 (t)
Delta BMI 6 (kg/m^2^)	10.11 ± 3.7	11.2 ± 15.3 (n = 70)	10.8 ± 12.8 (n = 103)	0.7 (t)
Delta BMI 12 (kg/m^2^)	10.8 ± 4.4 (n = 26)	13.1 ± 17.3 (n = 54)	12.3 ± 14.5 (n = 80)	0.5 (t)
HbA1c (mmol/L)	6.2 ± 1.1	6.1 ± 1.1	6.1 ± 1.1	0.9 (t)
TSH (mIU/L)	1.85 ± 1	2.3 ± 2.3	2.2 ± 2	0.3 (t)
Cortisol (µg/dL)	1.4 ± 2.3	1.1 ± 2.3	1.2 ± 2.3	0.7 (t)
Vitamin D (ng/mL)	18.1 ± 6.5	21.4 ± 9.2	20.4 ± 8.6	0.07 (t)
HTN	11 (33.3%)	26 (34.6%)	37 (34.3%)	0.9 (c)
T2DM	8 (24.24%)	16 (21.3%)	24 (22.2%)	0.7 (c)
CVA	0	0	0	-
CAD	1 (3%)	1 (1.3%)	2 (1.8%)	-
Afib	0	0	0	-
Depression	3 (9.1%)	5 (6.7%)	8 (7.4%)	0.7 (c)
EF (%)	64.98 ± 5.9	65.2 ± 6	65.1 ± 5.97	0.8 (t)
PASP (mmHg)	36.8 ± 9	40.2 ± 8.3	39.1 ± 8.6	0.11 (t)
CLO test	15 (45.5%)	32 (42.6%)	47 (43.5%)	0.8 (c)
Abnormal EGD	22 (66.6%)	38 (50.7%)	59 (54. 6%)	0.4 (c)
Normal EGD	11 (33.3%)	37 (49.3%)	49 (45.4 %)	0.4 (c)

**Table 2:** Types of surgery.

Type of surgery	Percentage
Robotic gastric sleeve resection	91/108 (84.26%)
Robotic Roux-en-Y gastric bypass	8/108 (7.41%)
Laparoscopic sleeve gastrectomy	6/108 (5.56%)
Revision	2/108 (1.85%)
Laparoscopic Roux-en-Y gastric bypass	1/108 (0.93%)

## Discussion

In eligible patients, bariatric surgery results in substantial weight loss. In a study on individuals between the ages of 20 and 64 in NHANES comparing individuals who had bariatric surgery with those who were eligible for it, but did not get surgery, it was found that post-bariatric surgery, there was a five times higher likelihood of achieving at least 20% weight loss. Participants who had surgery had a reduction of 15.5 BMI units and a 31% weight loss compared to 4.4 BMI units and 9.9% weight loss in the eligible but not operated group. Overall, 86.2% of the operated patients achieved 20% weight loss compared to 15.3% of the non-operated group [6]. Most patients in our study (84.26%) underwent robotic gastric sleeve resection.

Factors that have been implicated as being impactful in postoperative weight loss include age, race, socioeconomic status, gender, marital status, behavioral disorders, preoperative weight status, comorbidities such as DM, psychiatric illness, and sexual abuse, and institutional differences. Only a few are consistently supported in the literature [7].

In our literature review, we found a few studies that examined OSA as a predictor of post-bariatric surgery weight loss. In the larger of the studies by de Raaff et al., 816 patients were analyzed retrospectively. Of these, 522 (64%) had OSA, and 294 (36%) did not have OSA. The study aimed to evaluate the impact of OSA on % EWL at 12 months and BMI changes at 12 months after bariatric surgery. No information about ethnicity was available in the study. After adjustment for waist circumference, BMI, and age, no effect of OSA was seen on either the % EWL or BMI change at the end of 12 months [8]. In another study on a similar topic, Guggino et al. analyzed 371 patients included in a prospective cohort, the Severe Obesity Outcome Network cohort. Overall, 210 of these patients had moderate-to-severe OSA, and 161 had no OSA. Multivariable analysis of the data showed that age, initial BMI, and surgery type were associated with differences in % EWL. OSA independently was not associated with % EWL [9]. In a similar study by Mihalache et al., the authors analyzed a bariatric population that underwent laparoscopic sleeve gastrectomy. The primary outcome was % body fat change at 6 months and 12 months. The study revealed that patients with OSA had a lesser loss of body fat compared to patients without OSA. The weight loss experienced by both groups of patients was the same. All participants in this study were Caucasian [10]. In our study, we noted a similar lack of difference in the weight loss attained between patients with and without OSA at the end of 6 and 12 months.

Prevalence of DM ranges from 6.6% in patients without OSA to 28.9% in patients with severe OSA, thus showing an increase with severity [11]. OSA results in sleep fragmentation and intermittent hypoxemia, which, in turn, lead to increased sympathetic neural activity, oxidative stress, systemic inflammation, activation of the hypothalamic-pituitary axis, and alternation of circulating adipokines. These result in insulin resistance and beta-cell dysfunction culminating in diabetes. Even in patients without T2DM, the severity of OSA has been shown to be independently associated with insulin resistance. In reverse, the neuropathy that DM induces can affect the central control of respiration and upper airway neural reflexes. This can lead to the emergence of sleep apnea. Hence, the relationship between OSA and DM has been cited as being bi-directional [12]. Bariatric surgery results in an improvement in obesity and a drop in the apnea-hypopnea index. This is thought to be beneficial in controlling DM [12]. The American Society of Metabolic and Bariatric Surgery now recommends bariatric surgery for individuals with a BMI of 30-34.9 kg/m^2^ with metabolic disease including hyperglycemia that does not respond to non-surgical options [13]. In our study, no significant difference was noted in the HbA1C levels or the prevalence of DM in the two groups.

Hypothyroidism is associated with OSA. Deposition of mucoproteins in the upper airway resulting in upper airway obstruction, neuropathy-induced disruption of the regulatory control of pharyngeal dilator muscles, and respiratory center depression have been cited as possible causes of OSA in hypothyroidism. The prevalence of hypothyroidism in OSA is low (0.4%), but subclinical hypothyroidism seems to be more common in OSA (11.1%) [14]. TSH levels are positively correlated with BMI [15]. In our study, we noted that the mean TSH level in the OSA group was higher than in the non-OSA group, although the difference did not reach statistical significance (2.3 ± 2.3 vs. 1.85 ± 1).

A 2023 meta-analysis including 5,592 individuals from 18 observational studies found lower levels of vitamin D in patients with OSA compared to those without (18.2 ± 5.6 ng/mL vs. 23.2 ± 4.3 ng/mL). The effect was significant only in people with moderate-to-severe OSA, and not in mild OSA [16]. Similarly, another meta-analysis revealed a 35% higher prevalence of vitamin D deficiency in obese subjects [17]. Perception of low social acceptance leading to decreased outdoor activities and increased use of clothes to cover more body is one theory cited to explain vitamin D deficiency in the obese. Sequestration of cholecalciferol by excess fat and greater local use of 25(OH)D are other theories in obese individuals [17]. Hypoxia-induced OSA is related to vitamin D deficiency via hypoxia-inducible factor 1 alpha [16]. There was no significant difference in the vitamin D levels between the two groups in our study. One of the possibilities for this could be the smaller number of severe OSA patients in our study.

In many centers, EGD is routinely performed before bariatric surgery, the rationale behind this being that the bariatric procedure might alter the foregut anatomy. Gomez et al. analyzed 232 patients cleared to undergo bariatric surgery and noted abnormal findings in 61.6% of patients, with 15.1% requiring medical management alterations, and 1.7% requiring alterations in surgical management. The most common finding on EGD was a small hiatal hernia (23.7%) [18]. In our study, the most common EGD finding was erythematous mucosa.

A few studies have demonstrated an association between OSA and *H. pylori*, whereas others have not [19-21]. In our study, the prevalence of *H. pylori* infection was found to be 43% in the total cohort, with no statistically significant difference between the two groups. However, this is higher than the average *H. pylori* prevalence of 36% in the country [22].

OSA is linked with pulmonary hypertension (PH) [23]. The mean pulmonary artery pressure increases during OSA. OSA patients perform a series of inspiratory efforts against a completely obstructed upper airway. These can last from 10 seconds to 2 minutes with negative pleural pressures that can go up to -60 mmH_2_O before resumption of ventilation. Such sleep-related events result in hypoxemia, hypercapnia, variations in intrathoracic pressure, and arousals associated with sympathetic upswings. The consequent changes in vascular tone and cardiac output modify the pulmonary artery pressure. PH due to lung diseases is classified as group 3 PH [24]. Independent of OSA, obesity has been linked with PH as well [25]. Two parameters were recorded on echocardiography in our bariatric population, i.e., EF and PASP, both showing no significant difference.

OSA is a known risk factor for HTN [26], T2DM [11], CAD [27], CVA [28], Afib [29], and MDD [30]. Differences were not noted in any of these diseases.

The study has certain limitations. Behavioral changes such as an increased sedentary lifestyle and reduced physical exertion as a result of the coronavirus pandemic might have affected the findings of the study. This was a single-center study. Postoperative data was available only for weight and BMI, and not for other metabolic parameters such as HbA1C, TSH, cortisol, and vitamin D.

We noted the following major strengths of this study: the majority of the patients in this study were Hispanic. The population was predominantly from the inner-city area, which is underserved and has traditionally not been well studied.

## Conclusions

In an inner-city population comprising mostly Hispanic patients who qualified for bariatric surgery, no significant differences existed either in preoperative anthropometric or laboratory characteristics between patients with or without OSA. Bariatric surgery led to substantial weight loss in this population in OSA and non-OSA patients. Postoperatively, there was no difference in the amount of weight loss achieved between the OSA and non-OSA groups.
